# Alternative High-Performance Fibers for Nonwoven HEPA Filter Media

**DOI:** 10.1007/s41810-022-00161-6

**Published:** 2022-10-18

**Authors:** Ivan P. Beckman, Gentry Berry, Heejin Cho, Guillermo Riveros

**Affiliations:** 1 Information Technology Laboratory, U.S. Army Engineer Research and Development Center, Vicksburg, MS 39180 USA; 2 Institute for Clean Energy Technology, Mississippi State University, 205 Research Blvd., Starkville, MS 39759 USA

**Keywords:** Air filtration, HEPA filter media, Electrospinning, Melt-blowing, Ceramic, Carbon, Glass, Fibers, Cellulose, Keratin, Regenerated, ASME AG-1

## Abstract

Continual research, development, and advancement in air filtration technology is important to abate the ever increasing health hazards of air pollution and global pandemics. The purpose of this review is to survey, categorize, and compare mechanical and thermal characteristics of fibers to assess their potential applicability in air filter media. The history of high-efficiency particulate air (HEPA) filter development explains how we arrived at the current state of the art nonwoven fibrous borosilicate glass filter paper. This review explores the history and practical uses of particular fiber types and explains fiber production methods in general terms. The thermal and mechanical properties of particular fibers are examined using the codes and standards produced by the American Society of Mechanical Engineers (ASME) to generalize the applicability of fiber categories for HEPA filter units within the nuclear air cleaning industry. This review discusses common measurements for specific strength and tenacity used by the textile and construction industries. Particular fibers are selectively compared for density, tensile strength, tensile stiffness, flexural rigidity, moisture regain, decomposition temperature, and thermal expansion. This review concludes with a subjective assessment of which types of fibers may be appropriate to study for HEPA filtration.

## Introduction

Air filtration is vital to sustaining life. In 2016, the World Health Organization (WHO) estimated between seven and eight million people globally die from poor air quality and air pollution (United Nations Environment Programme 2021). Today more than 92% of the world’s population lives in areas that exceed dangerous levels of particulate aerosol from both indoor and outdoor sources (United Nations Environment Programme 2021). Surgical masks and N95 masks for personal protection were commonplace throughout 3 years of the global SARS-COVID19 pandemic. The United Nations Resolution 70/1, Transforming our world: the 2030 Agenda for Sustainable Development, set a goal for reducing the number of deaths worldwide caused by air pollution and contamination (General Assembly resolution 2030). The 2019 UN Blue Skies Campaign clearly linked the negative effects of air pollution to human health, longevity, and climate change. General Assembly Resolution 74/212 of 2019 set September 7^th^ as Clean Air Day and recognized, “Clean air is important for the health and day-to-day lives of people, while air pollution is the single greatest environmental risk to human health and one of the main avoidable causes of death and disease globally” (United Nations General Assembly Resolution 2020).

The development and advancement of high-efficiency particulate air (HEPA) filters demonstrate efforts toward sustain clean breathable air. The U.S. Department of Energy (DOE) installs HEPA filters as a line of defense to contain hazardous particles in high-level waste (HLW) storage tanks. For many years Lawrence Livermore National Laboratory (LLNL) has conducted extensive research on better materials and methods to filter and clean the ventilation air for HLW storage tanks (Bergman and Sawyer 1988; Bergman et al. 1996; Mitchell et al. 2012; Adamson 1998). HEPA filters are common today, however, HEPA filter technology has seen only minor advancement over the past several decades. The most common filtration media in use today, glass fiber filter paper, is fragile and prone to fire and water damage, as demonstrated by the Rocky Flats fire of 1957 (Ackland 1999). While better than the original cellulose–asbestos fibrous media, glass fibrous media performs poorly in high temperature applications and high humidity environments. The Defense Nuclear Facilities Safety Board (DNFSB) highlighted the need for alternative nonflammable HEPA filters (Conway 1999).

Recent development of new materials and production techniques suggests better materials and designs may enable advancement of air filtration technology. Currently most personal protective masks are constructed with polypropylene fibers, while most industrial filter media are produced with pleated glass fiber paper. Alternative fiber types should be explored for inclusion in air filtration media, to continue the advancement of both safety and performance of HEPA filters. The U.S. Customs and Border Protection agency maintains a list of 1200 fiber trade names that represent 43 generic fiber categories, acknowledging that the list is neither exhaustive nor complete (U.S. Customs 2021). The purpose of this review is to survey fiber types and compare mechanical, thermal, and chemical characteristics of particular fibers to advance air filtration technology and assist developers in the selection of fibers for air filter media. Special attention in this review is toward HEPA filter media intended for use by the nuclear air and gas industry.

## Background Information

### Brief History of HEPA Air Filtration

Lewis Haslett is credited with the first modern air filter respirator in 1849 with his “Lung Protector” invention and patent (Haslett 1849). Haslett developed his respirator with woolen fabric filter media. Ninety years later, at the outbreak of the Second World War, gas masks intended for smoke and dust filtration were still built with wool and cotton. The threat of chemical warfare brought new advancement in air filtration. The masks protecting German soldiers were produced from fine asbestos fibers and coarse esparto grass fibers and noted for exceptionally high-filtration efficiency and low airflow resistance to breathing (First 2022; Barnette and Smith 2003). British gasmask woolen filters suffered from clogging with oil-based smoke aerosols and did not perform as well as the asbestos fibrous media (Barnette and Smith 2003). The U.S. Army Chemical Warfare Services Command (CWS) in Edgewood Maryland tested the German filter materials and noted the superior performance of the asbestos fibers within the cellulose filter paper (Barnette and Smith 2003). From this experience, the CWS laboratory developed and produced “H-64” filtration paper which was later renamed “CWS type 6” paper and made from a combination of northern spruce sulfite, cotton, African esparto grass, and fine Bolivian Blue crocidolite asbestos fibers (First 2022; Barnette and Smith 2003). The laboratory noted the superior filtration performance of asbestos fibers cleaved to under 0.25 μm diameter (First 2022). The U.S. Army went on to produce “collective protector” filtration units that required more airflow than personal gasmasks, by deep-pleating CWS6 paper into square-framed canisters (Barnette and Smith 2003).

The Army’s collective protector filter units were adopted by the U.S. Atomic Energy Commission (AEC) in the late 1940’s to contain airborne radioactive particles in nuclear research facilities and ventilation exhaust systems of experimental nuclear reactors. To reduce dependency on foreign materials, the AEC replaced Bolivian crocidolite with Canadian asbestos. From 1944 to 1951, collective protector filter units were entirely made from all natural materials. The AEC began considering alternatives to cellulose–asbestos filter media in 1949 to overcome the flammability hazard at moderate and high temperatures. This led to the development of combined glass–asbestos fibrous media and all-glass fibrous media in 1951. The Naval Research Laboratory (NRL) developed methods to reduce the diameter size of glass fibers to 250 μm suggesting glass could be a substitute for asbestos fibers. The AEC type 1 filters were also termed absolute, super-interception, super-efficiency, and nuclear filters (First 2022; Barnette and Smith 2003). Humphrey Gilbert coined the acronym “HEPA” from a 1961 AEC report, “High-Efficiency Particulate Air Filter Units, Inspection, Handling, Installation” (Barnette and Smith 2003). The term HEPA today describes filter materials that achieve a high efficiency of 99.97% of 0.3 μm particles while maintaining a low resistance to air flow, measured by pressure drop across the filter material. The pleated borosilicate glass nonwoven fibrous filter paper media produced in 1951 is essentially still in production and use as HEPA filter units today.

### Fibers and Particulate Membranes

Air filters are commonly constructed with either fibrous media or porous particulate media (Hinds 1999). Porous membrane media is generally denser than fibrous filter media, which creates complex flow streamlines with high efficiency but also high air flow resistance. Porous media can be produced by a variety of methods with ceramics or sintered powdered metals. Composite air filter materials could be designed with both fibrous media and porous media, combining the advantages of both, for example gaining mechanical strength from a porous substrate layer while gaining high efficiency from a fibrous layer. A topic for further development is optimization of composite filter media with porous membranes and fibrous layers, however, the remainder of this review will focus on fibrous media.

### Air Filtration Theory

Up until the search for better protective filter materials during the Second World War, air filtration was thought to be similar to water filtration (Barnette and Smith 2003). Nobel Laureate Irving Langmuir is credited with initial development of modern air filtration theory based on particulate retention on fibers (Barnette and Smith 2003). Aerosol particles flowing through a filter media which come into contact with fibers stick to the fibers by electrostatic forces including Van der Waals forces. Langmuir’s air filtration theory was based on particle contact and retention from interception and diffusion mechanisms (Barnette and Smith 2003). Additions to his theory by Ramskill, Anderson, and many other researchers included the effects of inertial impact, gravitational settling, and electrostatic attraction (Barnette and Smith 2003). The individual particle capture mechanisms were consolidated into a single fiber efficiency (SFE) model by Davies in 1951 to integrate the combined effects of the many capture mechanisms (Hinds 1999; Davies 1973; Brown 1993). The SFE model is covered well throughout literature. Very small particles less than 0.3 μm in diameter are predominantly collected by Brownian diffusion, while particles larger than 0.3 μm are predominantly collected by interception and inertial impaction. Equation 1 consolidates the effects of multiple collection mechanics into a single fiber efficiency ${E}_{\Sigma }$ where ${E}_{\mathrm{R}}$, ${E}_{\mathrm{D}}$, ${E}_{\mathrm{DR}}$, ${E}_{\mathrm{I}}$, ${E}_{\mathrm{G}}$, and ${E}_{\mathrm{E}}$ are the single fiber efficiencies for interception, diffusion, interception of diffusing particles, inertial impaction, gravitational settling, and electrostatic forces, respectively.

1 $${E}_{\Sigma }=1-(1-{E}_{\mathrm{R}})(1-{E}_{\mathrm{D}})(1-{E}_{\mathrm{DR}})(1-{E}_{\mathrm{I}})(1-{E}_{\mathrm{G}})(1-{E}_{\mathrm{E}})$$

Of utmost importance is fiber diameter. Smaller diameter fibers have greater surface area to volume ratio. For example, by reducing the diameter by half, four smaller fibers now have equal volume of the larger fiber and four times the surface area. The increase in surface area for the same solid volume fraction is beneficial to filtration efficiency while detrimental to air flow drag. Fiber diameter ${d}_{\mathrm{f}}$, fiber packing density ${\alpha }_{\mathrm{f}}$, and filter thickness $t$ are three important parameters that combine with the single fiber efficiency ${E}_{\Sigma }$ in the calculation of total filter efficiency ${E}_{\mathrm{F}}$ shown in the following equation:

2 $${E}_{\mathrm{F}}=1-\mathrm{exp}\left(\frac{-4{\alpha }_{\mathrm{f}}{E}_{\Sigma }t}{{\pi d}_{\mathrm{f}}}\right)$$

The air flow resistance described by the pressure drop across the filter $\Delta P$ can also be related to the filter thickness, fiber diameter, a function of the solidity $f\left({\alpha }_{\mathrm{f}}\right)$, along with the air velocity ${U}_{0}$ and air viscosity $\eta$ as shown in Eq. 3. The basis of this analysis is Darcy’s Law. Various authors have developed independent functions of the solidity $f\left({\alpha }_{\mathrm{f}}\right)$ to fit pressure drop predictions to experimental observations.

3 $$\Delta P=\frac{\eta t{U}_{0}f\left({\alpha }_{\mathrm{f}}\right)}{{d}_{\mathrm{f}}^{2}}$$

The two objectives of air filtration are to increase the total filter efficiency while decreasing the resistance to airflow. The comparison of these two competing objectives is known as the “quality factor” or “figure of merit” of the filter, calculated as shown in Eq. 4 where $\mathrm{QF}$ represents the filter quality factor.

4 $$\mathrm{QF}= \frac{-\mathrm{ln}\left(1-{E}_{\mathrm{F}}\right)}{\Delta P}$$

### Definition of a Fiber

While fiber cross sections have various shapes, fibers are generally distinguished from other materials as having a large aspect ratio of length to cross sectional diameter, typically in excess of 100:1. The characteristics of the wide range of raw materials used to make fibers determine what fiber types are useful, including long continuous fibers and short cut fibers. Short cut fibers are further classified as chopped strands, whiskers, or staple fibers depending on their method of production. Metal filaments with diameters greater than 100 μm are generally considered wires, while diameters less than 100 μm are considered metal fibers.

### Fiber and Nonwoven Media Production Techniques

There many techniques to produce fibers, and many techniques to produce nonwoven media from fibers (Henning et al. 2021). Basic methods of nonwoven fibrous media production include dry laying (including air-laid and carded), wet laying, spun-lanced, spun-bonded, melt-blown, thermal bonded, needle-punched, and electrospun (Wang 2008; Kellie 2016). Fibers used in air filtration media can be classified as short (typically 1 mm to 20 mm) or long (greater than 20 mm). Dry-laid and wet-laid techniques use shorter fibers which are cut or produced at a target length, dispersed in air or water, and deposited into a mat to form nonwoven fibrous media (Wang 2008). Carding is a technique traditionally used in cotton production to mechanically disentangle, separate, and intermix fibers into a continuous mat. Needle punching or felting are techniques to bond short cut fibers into a nonwoven mat. Melt-blown, spun-bonded, and electrospun techniques have the ability to produce long fibers with small diameters. Electrospinning easily produces fibers with diameters in the 50 to 250 nm range. Melt-blowing produces fibers with diameters around 5 um, and spun-bonded fibers range in the 20 μm range (Wang 2008).

“Spinning” describes the method of fiber production, while “laying” describes the method to produce nonwoven media from fibers. Spinning represents the extrusion of a liquid material through a spinneret nozzle or needle to produce continuous fibers (Luo et al. 2012). If the liquid material is prepared for spinning by melting, the process is known as melt-spinning, which includes melt-blowing techniques. If the material is prepared for spinning by dissolution, the technique is known as solution spinning, which can be further categorized into dry-spinning, wet-spinning, dry–wet-spinning, sheared wet-spinning, and gel-spinning. Electrospinning uses a high voltage electric field to eject fibers from the liquid, and can be applied to both solution spinning and melt-spinning. Other methods to produce fibers and whiskers for air filtration media include chemical vapor deposition (CVD), bundle drawing, foil shaving, and machining.

Bundle drawing is a technique developed in the 1930’s to produce malleable metallic fibers. Thousands of metal wires are bundled together and drawn through reducing dies to decrease the bundle diameter and the diameter of each individual fiber. The bundles are then grouped into larger numbers and redrawn, resulting in smaller hexagon-shaped fibers. Recent advances in bundle drawing have resulted in metallic fibers in the range of 200 nm and smaller (Bekaert 2020). To produce nonwoven media, bundle drawn wires must be chopped to size and assembled through dry-laid or wet-laid techniques.

### Characteristics of Fiber Types

Fibers are organized and classified for comparison differently by the construction industry and textile industry. The textile institute publishes a fiber classification chart which first separates fibers into natural and man-made categories (Denton and Daniels 2002). Natural fibers are those that occur in nature, to include fibers produced by plants, animals, and minerals. Another simple classification is whether the fibers are made from organic or inorganic materials. Organic fibers can be further categorized into synthetic polymer, natural cellulose, regenerated cellulose, keratin, and carbon fibers. Natural plant-based fibers are made with the protein cellulose, while animal-based fibers are made from the protein keratin. If the plant is harvested, dissolved, then synthesized into fibers, the term “regenerated” cellulose describes a dual synthetic-natural category. Inorganic fibers can be subdivided into ceramic, oxide, non-oxide, elemental, mineral, metal, and volcanic rock, as in basalt fibers.

#### Natural Plant-Based Cellulose Fibers

Wood pulp, derived from both hardwood (oak, gum, birch, beech, aspen, eucalyptus) and softwood (pine, spruce, fir, cedar, hemlock, redwood), is a common material in nonwoven fibrous filtration paper media (Hutten 2015). Wood chips are typically cooked into pulp through the ‘kraft’ process, which uses sodium hydroxide, sodium sulfide, sulfurous acid, and bisulfite to break down the lignin at 170 °C to create wood pulp. Wood fibers are then bleached, fibrillated, and wet-laid to form filter paper (Hutten 2015).

Natural plant-produced fibers other than those produced by trees are also called ‘vegetal’, ‘vegetable’, ‘plant’, ‘cellulosic’, and ‘lignocellulosic’ fibers to name a few names (Siqueira et al. 2010). Cotton is a common cellulosic fiber made up of 99% pure cellulose. The cotton gin mechanically removes short fibers known as linters from harvested cotton. Cotton staple and first-cut linters can be made directly into air filtration material through a needle felting or needle punching process where nonwoven fibers are mechanically interlocked with special barbed needles that bonds fibers together. First-cut and second-cut linters can also be made into filtration media through a wet-laid process similar to wood pulp. Other common forms of cellulose fibers include abaca, hemp, esparto grass, sisal, jute, kenaf, and flax. Vegetal fibers are harvested from all parts of vegetable plants, and can be categorized into seed, stem, leaf, or husk as shown in Table 1.

The relative global abundance and ease of harvesting natural cellulose fibers make them attractive candidates for air filtration. Cotton materials have significant use as filtration media for personal protective face masks, typically as outer layers for an inner layer of polypropylene fiber material. In addition to cotton, kapok fibers have seen increased interest and research in the filtration of oil-based aerosols (Sun et al. 2018). The bast fibers flax, jute, hemp, and ramie continuously undergo study for their usefulness in air filtration (Mandal and Srimani 1987; Jianyong and Jianchun 2015; Manaia et al. 2019; Small and Marcus 2002; Anandjiwala and Boguslavsky 2008; Jose et al. 2016). Esparto grass fibers were used in early HEPA filter development, while sisal and abaca fibers have similarly attracted attention and study for their potential in air filtration (Lawrence et al. Jun. 2015).

#### Regenerated Cellulose Fibers

Regenerated cellulose fibers are considered both natural and synthetic categories, since the basic cellulose is derived from harvested plant-based materials and synthesized into fibers. The first production of regenerated cellulose synthetic fibers is credited to Count Hilaire de Chardonnet in France in the early 1880s, receiving a patent on the process in 1885 (Morgan 1981). The American Viscose Company produced regenerated cellulose fibers starting in 1910, while the Dupont Fibersilk Company started production in 1921 (Morgan 1981). Today, viscose is a common term given to regenerated fibers that refers to the sodium hydroxide and carbon disulfide solution used to derive the cellulose. Rayon is a common term which replaced the initial description of synthetic silk in 1924 (Morgan 1981). Viscose fibers were a common filtration media in the past, and still useful in automotive air filtration, however, most home pleated air filters are now made from fully synthetic polypropylene.

#### Natural Animal-Based Keratin Fibers

Keratin is a natural protein with a fibrous structure produced by animals and found in hair, feathers, wool, horns, claws, and hooves. Wool was among the original filter materials of choice during the Second World War, and is still used as a common filter material for vacuum cleaner bags today (Hutten 2015). Animal-based keratin protein fibers are categorized and grouped as wool, hair, and silk as shown in Table 2.

**Table 1:** Vegetal fiber types and examples

Seed	Stem (Bast)	Leaf	Husk/fruit
Cotton, kapok, milkweed	Flax, hemp, jute, ramie, kenaf, kudzu, linden	Esparto, sisal, abaca, palm, manila, curaua	Coconut (coir), banana, agave

**Table 2:** Keratin fiber types and examples

Wool	Hair	Silk
Sheep, alpaca, Angora rabbit, American bison, cashmere goat	Horse, camel	Spider silk, silkworm silk

Sheep’s wool in particular has attracted interest in indoor air pollution abatement, including separation of formaldehyde from breathable air (Wang 2014). Silk proteins can also be regenerated through an electrospinning process to produce environmentally friendly and efficient air filter materials to reduce indoor air pollution (Min et al. 2018). Additional research into the application of natural wood, vegetal, and animal-based fibers as air filter media might contribute to air filtration technology.

#### Synthetic Polymer Fibers

Nylon, the tradename for polyamide 6–6 fibers, was the first wholly synthetic polymer fiber credited to Carruthers and Hill in 1932 and produced in the United States by Du Pont since 1935 (Morgan 1981). Paul Schlack of I. G. Farbenindustrie first polymerized caprolactum to polyamide-6 fibers in 1938, which were later produced commercially by the Allied Chemical Company in 1955 and known as Perlon fibers (Morgan 1981).

Polyester fibers were first produced shortly after polyamide fibers by the Calico Printers Association in England in 1940 under the name Terylene (Morgan 1981). Du Pont Company in the United States began research into polyester fibers in 1945 and produced polyethylene terephthalate (PET) fibers from dimethyl terephthalate and ethylene glycol under the tradename Dacron (Morgan 1981). Polyester fibers became popular textile blend fabrics worldwide.

Acrylic fibers by definition are composed of at least 85% of the polymer acrylonitrile, while modacrylic fibers contain between 35 and 85% acrylonitrile (Morgan 1981). Acrylic and modacrylic fibers were produced by Du Pont, Monsanto, Union Carbide, and Eastman companies starting in the 1950s and carried brand names of Orlon, Acrilan, Vinon, Dynel, and Verel (Morgan 1981). Polyacrylonitrile is easily produced in small nanometer scale diameter sizes through electrospinning, and is a popular precursor fiber for conversion to carbon fibers.

Olefin fibers, specifically polypropylene fibers, were first produced through discoveries by Karl Ziegler in Germany and Julio Natta in Italy in 1954 (Morgan 1981). Compared to all synthetic fibers, polypropylene is the least expensive to produce, while it is uniquely hydrophobic, resistant to chemicals, and stable in air and sunlight (Morgan 1981). The low melting point of polypropylene in particular and olefin fibers in general is a major drawback for use in HEPA air filtration applications. However polypropylene is the most popular fiber used in personal protective filter masks and air filters for home and industrial use where temperature range where low melting temperatures are acceptable. High-density polyethylene (HDPE) and ultra-high molecular weight polyethylene (UHMWPE) are olefin fibers with the highest strength-per-weight ratio of all fibers discussed in this review, and would perform well as HEPA air filter fibers if they had a higher melting point. Table 3 categorizes synthetic common synthetic polymer fiber types with trade name examples.

**Table 3:** Synthetic polymer fiber types

Amide	Polyester	Liquid crystalline	Olefin	Common polymers
Nylon 6, Perlon, Nylon 6.6	PET, Dacron, Terylene, Kodel	Para-aramid, meta-aramid, aromatic heterocycle, copolyester	Polypropylene, polyethylene, HDPE, LDPE, UHMWPE	PVA, PTFE, polyurethane, acrylic, modacrylic, polyacrylonitrile

#### Carbon Fibers

Carbon fibers are formed through heat treatment of precursor materials. Common precursors include polyacrylonitrile, various forms of natural and regenerated cellulose, and petroleum or pitch products. Laboratory tests have shown potential benefits of using carbon fibers in air filtration. Carbon nanotubes combined with activated carbon fibers show great potential for adsorbing ozone while also filtering particulate matter in indoor environments (Yang et al. 2017). Activated and ionized carbon fibers were tested for indoor air filtration with good results. Carbon nanotubes were also noted for their high efficiency and low pressure drop during filtration in the free molecular flow region (Li et al. 2014).

#### Ceramic Fibers

Initial attempts at ceramic fibers for air filtration date back to the Hulrburt Paper Company and Hollingsworth and Vose Company in the mid-1950s as they produced filter paper from Fiberfrax fibers made of silicon oxide-aluminum hydroxide (Barnette and Smith 2003). Ceramic fibers show high potential for achieving HEPA level air filtration. Laboratory production and testing of aluminum oxide-stabilized zirconium oxide fibers resulted in high filtration efficiency and low flow resistance (Jia et al. 2020). In addition to high efficiency and low resistance, the lightweight ceramic air filtration paper produced through blow spinning showed excellent thermal and mechanical properties with high flexibility, foldability, temperature range, and burn resistance (Jia et al. 2020).

#### Glass and Quartz Fibers

Melt-blowing or melt-spinning are common methods to produce small diameter glass fibers. Glass fiber filter paper, initially produced and tested by NRL as a substitute for asbestos fiber paper in the 1950s, continues to be the media of choice for deep pleated HEPA filter units today. Glass fibers are produced from many varieties of glass, which is categorized based on the ingredients that accompany silicon dioxide (silica). Quartz fibers are fully crystalized silica. Two common quartz fibers are Quartzel and Astroquartz. Quartz fibers have attractive qualities for air filtration, however, they are expensive to produce.

#### Mineral Fibers

Asbestos is a naturally occurring long thin fibrous material formed on silicate minerals. Asbestos fibers are divided into two classifications: serpentine (curly fibers) and amphibole (needle-like fibers). Chrysotile is the only form of serpentine asbestos, while amphibole asbestos includes amosite, crocidolite, tremolite, anthophyllite, and actinolite, as shown in Table 4. Chrysotile is the most common form of asbestos found in buildings built before 1980.

**Table 4:** Asbestos fiber types (Beckman et al. 2021)

Serpentine asbestos	Amphiboles asbestos
Chrysotile	Amosite (asbestos grunerite), crocidolite, tremolite, anthophyllite, actinolite

Asbestos fibers have significant resistance to heat and make excellent thermal and electrical insulation. Asbestos fibers were used in air filtration since the Second World War and up until discovery of the health risks of asbestos particles. Asbestos was a primary source of early HEPA filtration (Barnette and Smith 2003). In the 1970s, the health hazards caused by airborne asbestos fibers surfaced, and the use of asbestos fibers is now banned in many countries. Airborne asbestos fiber particles can become lodged deep into lungs and cause cancer. Asbestos fibers are no longer considered for HEPA filter media nor general construction because of the significant health risks.

## Mechanical, Thermal, and Chemical Considerations for Fiber Selection

The mechanical and chemical properties of air filtration media play an important role in the overall performance of an air filter. Characteristics of the fiber surface determine the ability of the fiber to contact and retain particles. Fiber tensile and flexural strength are important to prevent tearing of the filter media under variable flow conditions. The chemical properties of the media determine whether it will oxidize or decompose over time, and whether the media will combust or degrade under high temperature conditions.

### ASME AG-1 Specification

Since 1971 the American Society of Mechanical Engineers (ASME) has developed the code and standards to evaluate materials proposed for use in the nuclear air and gas industry. The initial ANSI N45.8 Committee was reorganized into the Committee on Nuclear Air and Gas Treatment in 1976 and produces the ASME AG-1 code for nuclear air and gas treatment. The AG-1 specifies requirements for materials testing and approval of nuclear grade HEPA filter media (ASME 2020). Section FC of the AG-1 serves as a useful guide to evaluate desirable physical and chemical properties important in air filtration media, to include tensile strength (under normal, heated, wet, and irradiated conditions), water repellency, combustion resistance, flexibility, and mildew resistance. Airflow resistance and particle filtration efficiency are two basic parameters to qualify as HEPA filter media. The maximum allowable pressure drop across the filter media is 320 Pa tested at ambient conditions with minimum face velocity of 2.5 cm/s. The filter media must have an efficiency of at least 99.97% when tested with 0.3 μm diameter particles and an efficiency of at least 99.9% when tested with the most penetrating particle size at 2.5 cm/s. The ASME AG-1 standards show a preference for noncombustible, water repellant, and mildew resistant fibers that have high strength, toughness, and flexibility shown in Table 5.

**Table 5:** Parameters and specifications of HEPA filter media from ASME AG-1

Mechanical	Chemical	Thermal	Environmental conditions
Tensile strength Flexural strength Flexibility	Water repellant Mildew resistant Non-combustible Corrosion resistant	Temperature range Burn resistance	Temperature Wet & humid Irradiated

### European and International Specifications

Since 1998, the European Committee for Standardization define H13 and H14 HEPA filter media as having an efficiency of 99.95% and 99.995% respectively with “High Efficiency Air Filters” standard EN 1822 (Committee for Standardization 2022). The International Organization of Standards adopted the European EN 1822, and added categories to match other international standards in existence, with the publication of “High Efficiency Filters and Filter Media for Removing Particles from Air,” ISO 29463 (International Organization for Standardization 2022). The ASME AG-1 specification for nuclear grade HEPA filters compares with the European and ISO standards as shown in Table 6. Notable differences include the air filtration nominal flow rate and particle size. The EN 1822 and ISO 29463 efficiency standards are based on the most penetrating particle size (MPPS), while the ASME AG-1 standard specifies both MPPS and 0.3 μm particle sizes.

**Table 6:** Comparison of HEPA specifications for nonwoven fibrous media

ISO 29463	EN 1822	ASME AG-1	Efficiency (%)	Flow rate (cm/s)	Particle size	Resistance (Pa)
35H	H13	–	99.95	5.3	MPPS	–
–	–	HEPA	99.97 / 99.9	2.5	0.3 μm / MPPS	320
40H	–	–	99.99	5.3	MPPS	–
45H	H14	–	99.995	5.3	MPPS	–

### Fiber Diameter Sizes

**Fig. 1:**
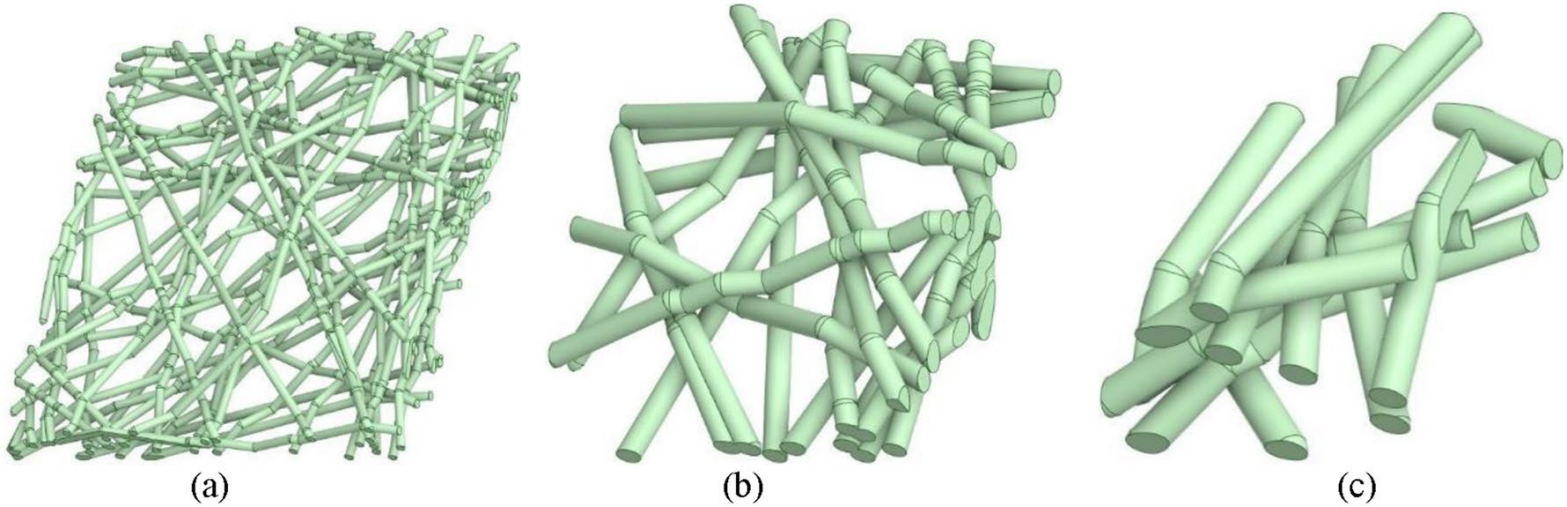
Comparison of filtration media produced from a range of diameter sizes. **a** 0.1 μm Diameter fibers. **b** 1.0 μm Diameter fibers. **c** 2.0 μm Diameter fibers

Fiber diameter size is a critical parameter that affects filtration efficiency and flow resistance of an air filter. Smaller diameter fibers have a greater ratio of surface area to volume and generally result in higher filtration efficiency. The SFE model is useful in illustrating the effects of various fiber diameter sizes on filtration efficiency and flow resistance. Figure 1 illustrates three digital twin replica geometry of air filter media of varied diameter sizes, each with a consistent face coverage of 100%. Here face coverage is defined as the ratio of summed area of the fibers projected onto the plane perpendicular to airflow compared to total area, which can be thought of as the shadow the fibers on the plane normal to flow.

**Fig. 2:**
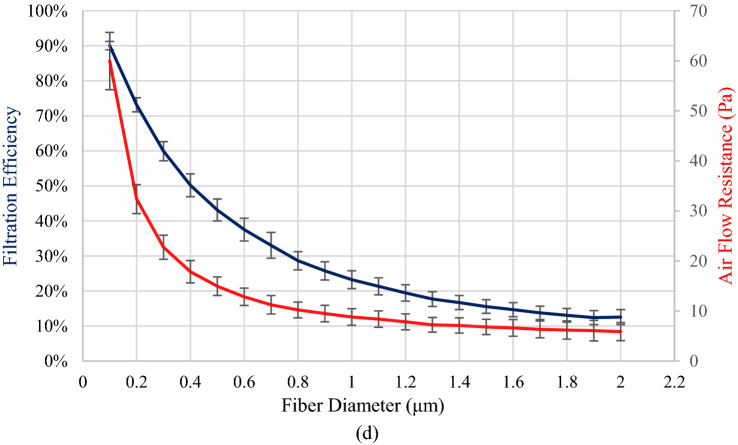
Resulting filtration efficiency and flow resistance. Error bars on filtration efficiency and air flow resistance represent 95% for 100 observations

Figure 2 shows the resulting effects on air filtration efficiency and air flow resistance of varying fiber diameters from 1 to 20 μm, while maintaining consistent face coverage of 100%. For this analysis, a digital twin replica of filter media was produced using a Python script and Ansys Spaceclaim using a procedure shown in an earlier work (Beckman et al. 2021). The thickness of the filter media varied from 1.32 to 11.28 μm, while the solidity varied from 6.24 to 15.35%.

While many regenerated and synthetic fibers are available in various diameter sizes, most natural cellulose and keratin based fibers are constrained by a range. The very small fiber diameters of carbon nanotubes, ranging from 1 to 5 nm, create a significant advantage in air filtration (Saifuddin et al. 2012). Electrospinning as well produces small diameter fibers in the range of 50–900 nm. Table 7 illustrates typical diameter ranges of select fibers. For comparison, the average size of a human hair is 50–70 μm.

**Table 7:** Comparison of example fiber diameter sizes

Fiber	Category	Typical diameter (μm)
Carbon nanotube	Carbon	0.001 to 0.005
Toyobo Zylon PBO	Synthetic polymer	1.2
Honeywell Spectra 1000 UHMWPE	Synthetic polymer	1.7 to 3.1
Alumina Saffil	Ceramic	3
E Glass (Alumino Borosilicate)	Glass	3 to 20
Toray T1100G carbon fiber (PAN)	Carbon	5
Fortisan Viscose Rayon	Regenerated cellulose	9
Quartzel fibers	Quartz	9 to 14
Basalt fibers	Basalt	10 to 20
Solvay P-25 carbon fiber (pitch)	Carbon	11
American uppers cotton fibers	Natural cellulose	11 to 22
Dupont Kevlar 49	Synthetic polymer	12
Oak wood fibers	Natural cellulose	13
Nylon 6 fibers	Synthetic polymer	14
Polyacrylonitrile	Synthetic polymer	16
Wool fibers	Natural Keratin	18 to 44
Polypropylene	Synthetic Polymer	38
Human hair for comparison	Natural keratin	50 to 70
SCS ultra silicon carbide fiber	Ceramic	75
Boron fiber	Mineral	142

### Fiber Density

Volumetric density and linear density are two measurements of fiber mass important for many extensive thermal and mechanical properties. Volumetric density, shown in Table 8, compares the mass of a fiber to its volume. Linear density similarly compares the mass to the fiber length. Over the past several 100 years, the textile industry developed the units of denier (de) and tex to compare and evaluate threads and yarns. One denier has a linear density of one gram of mass per 9000 m of fiber. The tex is similarly a measurement of one gram mass per 1000 m of fiber, and a decitex or dtex is one gram of mass per 10 km of fiber. Measurements of linear density become important when comparing the specific strength of fibers.

**Table 8:** Density of Fibers (Beckman et al. 2021)

Fiber type	Density (g/cm^3^)
Cellulose fibers	0.14 to 1.54
Polymer fibers	0.90 to 1.54
Keratin fibers	1.30 to 1.34
Regenerated cellulose	1.25 to 1.52
Carbon fibers	1.74 to 2.20
Glass and quartz fibers	2.10 to 2.60
Ceramic fibers	1.80 to 3.90
Mineral fibers	2.64 to 2.70
Metallic fibers	1.74 to 8.92

### Fiber Strength and Stiffness

The ASME AG-1 specifies a tensile breaking strength of HEPA filtration media must be at least 430 N/m in the machine direction and 350 N/m in the cross direction, with a maximum allowable elongation of 0.5% at rupture (ASME 2020). This standard ensures HEPA filters are not easily ripped or torn by accident and do not rip and tear when subjected to sustained high pressure conditions. The filtration media must maintain high strength as well during heated, wet, and irradiating conditions. In the cross-machine-direction, the media must maintain a tensile strength of 110 N/m after high temperature airflow of 370 °C for five minutes, a tensile strength of 170 N/m after soaking in water at ambient temperature for 15 min, and tensile strength of 170 N/m after receiving gamma irradiation for an integrated dose of 6.0E7 to 6.5E7 rad at a dosage rate not to exceed 2.5E6 rad/hr (ASME 2020). While tensile strength of filtration media under heated, wet, and irradiated conditions depends greatly on the composition and characteristics of binders and other materials in the filtration media, the performance of individual fibers is critical to overall strength. Table 9 shows tensile strength in units of GPa, and tenacity in units of MegaYuri (MY), which is described in detail below.

**Table 9:** Tensile strength and tenacity of fibers (Beckman et al. 2021)

Fiber family	Yield strength (GPa)	Tenacity (MY)
Carbon fibers	2.20 to 7.00	1.20 to 4.00
Glass and quartz fibers	2.00 to 6.00	0.78 to 2.72
Polymer fibers	0.33 to 5.80	0.27 to 3.80
Ceramic fibers	1.40 to 5.90	0.40 to 1.90
Mineral fibers	2.50 to 4.80	1.00 to 1.80
Metallic fibers	0.22 to 2.21	0.03 to 0.28
Keratin fibers	0.19 to 1.40	0.14 to 1.07
Regenerated cellulose	0.17 to 0.89	0.13 to 0.59
Cellulose fibers	0.07 to 0.89	0.12 to 0.59

### Fiber Tenacity (Specific Strength)

Typical measurements of material strength are given in units of force per cross sectional area, such as gigapascals. However, many fibers have irregular shaped cross sections and hollow cores making it difficult to accurately measure the cross sectional area and volumetric density. Specific strength measures the breaking force of a fiber compared to its linear density, which determines the tensile force required to break a fiber compared to its denier or tex. The textile industry calls this measurement the tenacity of the fiber. Common measurements of tenacity include newtons per tex $\left(\frac{\mathrm{N}}{\mathrm{tex}}\right)$, grams per denier (gpd), and pascals per volumetric density $\left(\frac{\mathrm{GPa}}{\mathrm{g}/{\mathrm{cm}}^{3}}\right)$. The international space elevator consortium advocated for a new unit “yuri” as a measure of specific strength for cable materials using SI base units. The equivalent cross sectional areas of strength and density cancel leaving the breaking force divided by linear density as shown in the following equation. Fiber tenacity has units of $\frac{{\mathrm{m}}^{2}}{{\mathrm{s}}^{2}}$.

5 $$\mathrm{yuri}\,=\frac{\mathrm{Pa}}{\frac{\mathrm{kg}}{{\mathrm{m}}^{3}}}=\frac{\frac{\mathrm{N}}{{\mathrm{m}}^{2}}}{\left(\frac{\mathrm{kg}}{\mathrm{m}}\right)\left(\frac{1}{{\mathrm{m}}^{2}}\right)}=\frac{\mathrm{N}}{\frac{\mathrm{kg}}{\mathrm{m}}}= \frac{\mathrm{N m}}{\mathrm{kg}}= \frac{\left(\frac{\mathrm{kg m}}{{\mathrm{s}}^{2}}\right)\mathrm{ m}}{\mathrm{kg}} = \frac{{\mathrm{m}}^{2}}{{\mathrm{s}}^{2}}$$

Breaking force is implicit in the textile industry measurement of grams per denier (gpd). Grams of mass multiplied by the acceleration of gravity reveals breaking force as gram-force newtons as shown in Eq. 6 which also shows the conversion between gpd and yuri. Equation 7 relates the measurement of newtons per tex to gpd and yuri, showing one mega yuri (MY) equivalent to one newton per tex and 11.33 gpd. The construction industry measures specific strength of fibers as the breaking strength of the fiber per volumetric density. The units work out such that one $\frac{\mathrm{MPa}}{\mathrm{g}/{\mathrm{cm}}^{3}}$ is equivalent to 1000 yuris, and one $\frac{\mathrm{GPa}}{\mathrm{g}/{\mathrm{cm}}^{3}}$ is equivalent to one $\frac{\mathrm{N}}{\mathrm{tex}}$ and one MY as shown in Eq. 8.

6 $$\mathrm{gpd}=\frac{\mathrm{g}}{\mathrm{den}}\approx \frac{\mathrm{g }\left(9.80665\frac{\mathrm{m}}{{\mathrm{s}}^{2}}\right)}{\frac{\mathrm{g}}{\mathrm{9,000 m}}}\approx \mathrm{ 88,260}\frac{{\mathrm{m}}^{2}}{{\mathrm{s}}^{2} }\approx\, \mathrm{88,260 yuri}$$

7 $$\frac{\mathrm{N}}{\mathrm{tex}}=\frac{\frac{\mathrm{kg m}}{{\mathrm{s}}^{2}}}{\frac{\mathrm{g}}{\mathrm{1,000 m}}}=\mathrm{1,000,000}\frac{{\mathrm{m}}^{2}}{{\mathrm{s}}^{2} }=\mathrm{MY}\approx 11.33\mathrm{ gpd}$$

8 $$\frac{\mathrm{GPa}}{\mathrm{g}/{\mathrm{cm}}^{3}}=\frac{\mathrm{1,000,000,000}\frac{\mathrm{N}}{{\mathrm{m}}^{2}}}{\left(\frac{\mathrm{kg}}{\mathrm{1,000}}\right)\left(\frac{\mathrm{1,000,000}}{{\mathrm{m}}^{3}}\right)}=\mathrm{ 1,000,000}\frac{{\mathrm{m}}^{2}}{{\mathrm{s}}^{2} }=\frac{\mathrm{N}}{\mathrm{tex}}=\mathrm{MY}$$

**Fig. 3:**
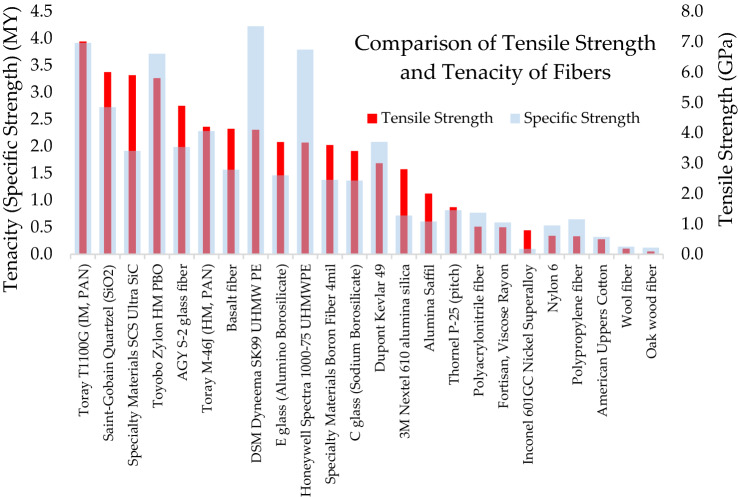
Comparison of tensile strength and tenacity of select fibers (Beckman et al. 2021)

The comparison of tensile strength and specific strength of fibers is shown by Fig. 3, with the stronger fibers on the left side of the chart. While polyacrylonitrile-based carbon fibers are the strongest overall fibers, the synthetic UHMWPE fibers have slightly higher tenacity because of the lower density of UHMWPE materials. Conversely, ceramic, glass, and metallic fibers have lower values of tenacity because they are composed of very dense material.

### Fiber Stiffness

**Fig. 4:**
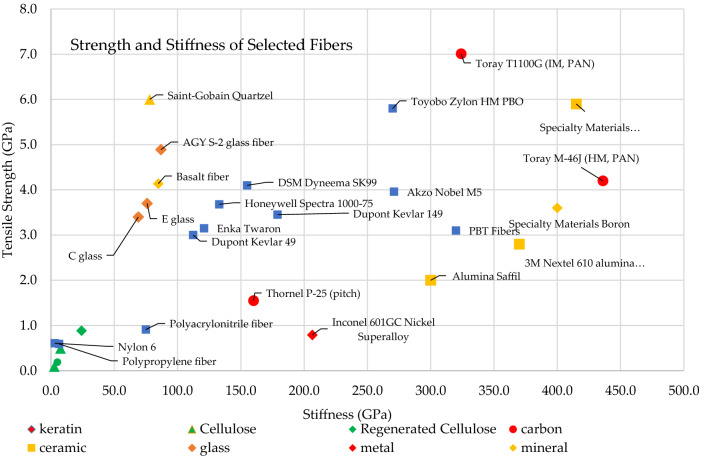
Comparison of tensile strength and stiffness of fibers (Beckman et al. 2021)

Fiber stiffness is a parameter that accompanies fiber strength in importance. Air filter materials must resist excessive strain when exposed to high pressure. Carbon, ceramic, and mineral fibers are the stiffest, while natural cellulose and keratin fibers have the least stiffness. Natural wood fibers, synthetic polymer fibers, and regenerated cellulose fibers are the most elastic. An overall comparison of fiber strength and stiffness is shown in Fig. 4.

### Fiber Flexibility and Flexural Rigidity

Fiber flexibility and flexural rigidity can be thought of as the respective ease or resistance to bending motion of the fiber. Fiber flexibility is important in air filter design. The ASME AG-1 specifies a standard for media flexibility, requiring the media to show no tears, breaks, cracks, or fiber separation after being drawn back and forth five times over a 4.8 mm mandrel to an arc of 180 degrees (ASME 2020). The filtration media must maintain the same filtration performance, with no more than 0.03% penetration by monodisperse 0.3 μm particles and no more than 0.1% penetration by the most penetrating particle size. This standard favors the selection of fibers that are tough yet flexible enough to bend repeatedly without breaking.

Fiber flexibility can be shown by the ratio $k/M$ where $k$ represents the inverse of the fiber radius of curvature, and $M$ is the moment of inertia. Equation (9) shows the inverse relationship between fiber flexibility, tensile modulus $E$, and fiber diameter $d$. In classical beam theory, flexural rigidity of a beam is given as $EI$, with $E$ in units of Pa and $I$ as the second moment of area in units of m^4^, resulting in units of N·m^2^. The textile industry measures flexural rigidity of fibers using classical beam theory as shown in Eq. (10) with $s$ as the fiber “shape factor,” $\rho$ the volumetric density, $c$ the linear density, and ${E}_{\mathrm{s}}$ the specific modulus ($E/\uprho$) of the fiber.

9 $$\frac{k}{M}=\frac{64}{{\pi Ed}^{4}}$$

10 $$EI=\frac{s{E}_{\mathrm{s}}{c}^{2}}{4\pi \rho }$$

**Fig. 5:**
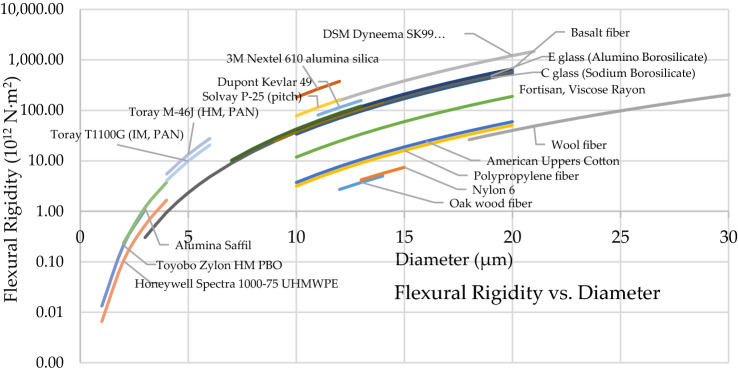
Flexural rigidity at actual diameter sizes

Taking into account realistic diameter sizes of each fiber type as shown in Table 7 above, Eq. 10 can provide a comparison of expected flexural rigidity, as shown in Fig. 5. Clearly fibers with larger diameters are rigid, and fibers with smaller diameters are flexible.

### Fiber Water Repellency and Mildew Resistance

Filter media must repel water in order to avoid problems in high humidity and wet conditions. The ASME AG-1 specifies the media must be prepared in accordance with the Technical Association of the Pulp and Paper Industry (TAPPI) standard T402, and tested for water repellency with procedure 125-8-1 Q101 (ASME 2020). To achieve the specification, the material must have an average water repellency of at least 5000 Pa. The degree of hydrophobicity of individual fibers is an important characteristic when selecting fibrous materials for filter media. The ASME AG-1 also specifies the filter media must be tested for mildew growth in accordance with MIL-STD 810 method 508 if required by the user.

**Fig. 6:**
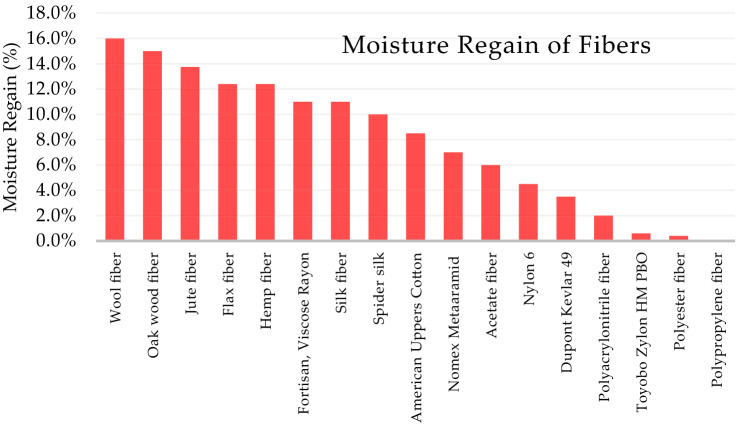
Moisture regain of selected fibers (Beckman et al. 2021)

Moisture regain indicates a fiber’s ability to absorb moisture, expressed as a percentage of water weight absorbed by the fiber compared to the dry weight of the fiber. Many organic fibers are hydrophilic and hygroscopic, meaning they have a strong affinity toward water and tend to absorb moisture from the air. Natural keratin and cellulose fibers absorb the most moisture. Wool fibers absorb nearly 17% of their dry fiber weight while silk fibers absorb approximately 11% moisture (Huson 2018). Wood fibers absorb nearly 15% of their weight as moisture. Cotton, jute, flax, and hemp absorb a significant amount of moisture from the air, as well do most regenerated cellulose fibers, which are also considered hygroscopic. Some but not all synthetic polymer fibers absorb moisture, depending on the properties of the synthetic material. Inorganic fibers, including ceramic, metallic, basalt, and glass, are nonhygroscopic and do not absorb moisture from the air. Figure 6 compares the moisture regain of selected fibers, showing mainly the fibers that will have significant moisture problems.

### Fiber Temperature Threshold and Fire Resistance

**Fig. 7:**
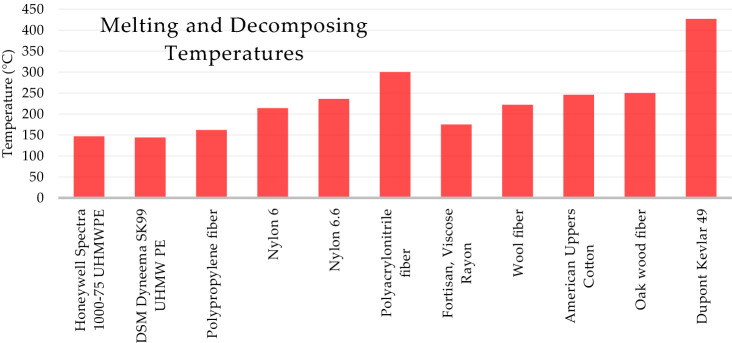
Comparison of melting and decomposing temperatures of select fibers

To pass ASME AG-1 specifications, the filter media must be noncombustible and must withstand high temperatures. The ASME AG-1 specifies that the combustible material in the filter media shall not exceed 7% by weight. This standard is tested by IEST-RP-CC021.1 by measuring the weight loss after subjecting the filtration media to elevated temperatures. The AG-1 also specifies the filter material must withstand a sustained pressure of 2400 Pa while subjected to continuous heated air flow of 370 °C. These standards ensure the filter is capable of sustained high efficiency filtration even when exposed to fire. The fibers shown in Fig. 7 have decomposing or melting temperatures lower than or near 370 °C. Cellulose fibers, keratin fibers, regenerated cellulose fibers, and synthetic polymer fibers are mostly unsuitable for HEPA filter units because of low temperature threshold. Ceramic, metallic, mineral, carbon, basalt, and glass fibers have temperature thresholds well above the 370 °C threshold. Zylon PBO is a synthetic polymer fiber with a reported degradation temperature of 650 °C which is above the threshold.

### Thermal Expansion and Contraction

**Fig. 8:**
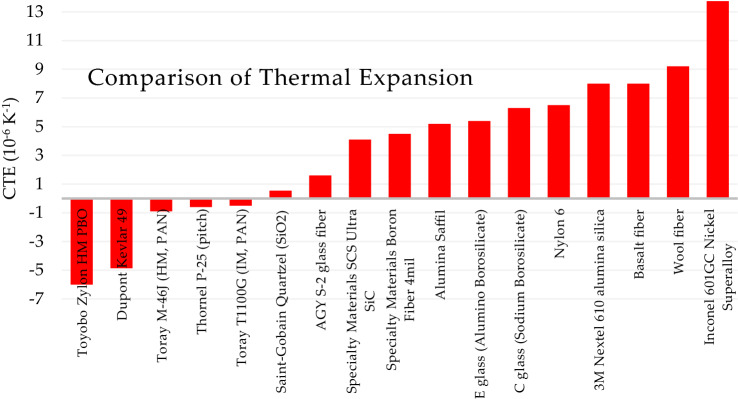
Comparison of thermal expansion (Beckman et al. 2021)

The coefficient of linear thermal expansion (CTE) is a material property that indicates the extent to which a material expands or contracts when the material changes temperature. Most fibrous materials have a slightly positive CTE which indicates they expand when heated and contract when cooled. Some fibrous materials, for example Kevlar and Zylon, have a negative CTE indicating contraction when heated and expansion when cooled. The disadvantage of using fibers in air filter media with a highly positive or highly negative CTE is the expansion or contraction changes the dimensions of the fibers, in turn affecting the air filtration and air flow resistance performance of the media. The advantage of carbon fibers, quartz fibers, and glass fibers is they have a very small CTE and are thus resistant to expansion or contraction when the filter media heats up or cools down. A comparison of thermal expansion coefficients for selected fibers is shown in Fig. 8.

### Overall Fiber Comparison

An overall subjective comparison of twenty four fibers is presented in Table 10 to guide the selection of fibers for research and development of potential alternative HEPA filtration media. The particular fibers listed in the table are meant to represent their general category of fibers, for example American Uppers Cotton fiber is intended to represent natural cellulose fibers, while Torray T1100 is intended to represent polyacrylonitrile-based carbon fibers. Melt-blown glass fibers are currently the dominant choice for HEPA filters based on ease of production, availability, and overall performance. Cellulose, regenerated, keratin, and most synthetic polymer fibers are unsuitable for HEPA consideration because of moisture or temperature requirements discussed above. In addition to other types of glass fibers, the fiber categories carbon, ceramic, mineral, basalt, and metallic are worthy of consideration for HEPA filtration.

**Table 10:** Overall fiber subjective comparison

Fiber	Category	Tensile strength	Tensile modulus	Bending	Moisture regain	Thermal expansion	Temp 370 °C
Cotton (Yan 2016)	Cellulose	Fair	Elastic	Flexible	Very High	Low	Decompose
Wood (Wikipedia 2021)	Cellulose	Fair	Elastic	Flexible	Very High	Low	Decompose
Rayon (Araújo 2011)	Regenerated	Fair	Elastic	Flexible	Very High	Low	Decompose
Wool (Huson 2018)	Keratin	Fair	Elastic	Flexible	Very High	Low	Decompose
Spectra, Dyneema (Honeywell 2021) (DSM 2021)	UHMWPE	High	Stiff	Moderate	None	Negative	Melt
Nylon 6/6.6 (MatWeb 2021)	Polymer	Fair	Elastic	Flexible	High	Low	Melt
Dupont Kevlar 49 (Dupont 2017)	Aramid	Moderate	Stiff	Moderate	Moderate	Negative	Decompose
Toyobo Zylon PBO (Toyobo 2021)	Polymer	Very High	Stiff	Rigid	Moderate	Negative	Near Max
Polyacrylonitrile (MatWeb 2021)	Polymer	Fair	Stiff	Moderate	Moderate	Low	Melt
Polypropylene (Araújo 2011)	Polymer	Fair	Elastic	Flexible	None	Low	Melt
Toray M-46 J (PAN) (Toray Composite Materials America 2022)	Carbon	High	Most Stiff	Most Rigid	None	Negative	Safe
Toray T1100G (PAN) (Toray Composite Materials America 2022)	Carbon	Very High	Stiff	Rigid	None	Very Low	Safe
Solvay P25 (pitch) (Solvay 2021)	Carbon	Fair	Stiff	Moderate	None	Very Low	Safe
Saffil (MatWeb 2022)	Ceramic	Moderate	Stiff	Rigid	None	Low	Safe
3 M Nextel 610 Al Si (3M 2022)	Ceramic	Moderate	Very Stiff	Rigid	None	Low	Safe
SCS Silicon Carbide (Specialty Materials 2021)	Ceramic	Very High	Very Stiff	Highly Rigid	None	Low	Safe
Quartzel, Astroquartz (Saint-Gobain 2022)	Quartz	Very High	Stiff	Moderate	None	Very Low	Safe
S2 glass fiber (AGY 2021)	Glass	High	Stiff	Moderate	None	Very Low	Safe
E glass (Jones and Huff 2018)	Glass	High	Stiff	Moderate	None	Low	Safe
C glass (Jones and Huff 2018)	Glass	Moderate	Stiff	Moderate	None	Low	Safe
D glass (Jones and Huff 2018)	Glass	Moderate	Stiff	Moderate	None	Low	Safe
Boron 4mil (Specialty Materials 2022)	Mineral	Moderate	Very Stiff	Highly Rigid	None	Low	Safe
Basalt (Militký et al. 2018)	Volcanic	High	Stiff	Moderate	None	Low	Safe
Inconel 601GC Nickel (MatWeb 2022)	Metal	Fair	Stiff	Rigid	None	Moderate	Safe

## Nonwoven Air Filtration Media

Many of the fibers listed in Table 10 are already in production as nonwoven media, primarily intended for structural reinforcing in composites but also potentially capable of air filtration. Table 11 shows examples of nonwoven fabric, paper, and matting.

**Table 11:** Trade names of non-woven fabric, paper, and matting (Starr 1995)

Fiber family	Nonwoven fabric, paper, and matting
Glass	Verlock, Textoglass, JPS, Nittobo, Orcoweb, SFT, Vetrotex
Carbon	Carbotex, Carboflex, Carrfibre, Graflok, Uniloc, Unilay, Layrite, Fibral, Technoglas, Fortafil, Panex, Torayca, Fibertec, Technimat, Kureha, Filacron
Metals	Bekinox, Bekipor, Brunsmet, Lantorine, Fensor, Fibrex
Oxidized PAN	Fortafil, Oxipan, Panox, Sigrafil, Avox, Pyromex, Panotex, Pyron
Aramid	Carrfibre, Nomex, Aralok, Fibertek
UHMWPE	Spectra, Dyneema, PBI, Tekmilon, Texxes, Kermel, Tenfor

### Glass Fiber Nonwoven Media

Most industrial HEPA filter units today are made with melt-blown borosilicate glass fiber pleated paper. Example producers of H14 filtration paper found online are shown in Table 12, with stated filtration efficiency, flow resistance, basis weight, thickness, and media tensile strength.

**Table 12:** Example producers of H14 HEPA filter media

	Guangzhou Clean Link (Guangzhou 2022)	Hebei Amusen (Hebei Amusen 2022)	Shandong Renfeng (Shandong Renfeng 2022)	Hebei Fangyu (Hebei Fangyu 2022)	CHMLAB (CHMLAB Group 2022)
Efficiency (%) at 5.3 cm/s	99.993	99.995	99.998	99.995	99.995
Resistance (Pa) at 5.3 cm/s	380	390	372	420	450
Basis weight (g/m^2^)	75	75	76	74	75
Thickness (mm)	0.50	0.35	0.40	0.33	0.35
Tensile strength MD (N/m)	980	1000	1000	1250	800
Tensile strength CD (N/m)	500	400	–	–	700

*MD* machine direction, *CD* cross direction

**Fig. 9:**
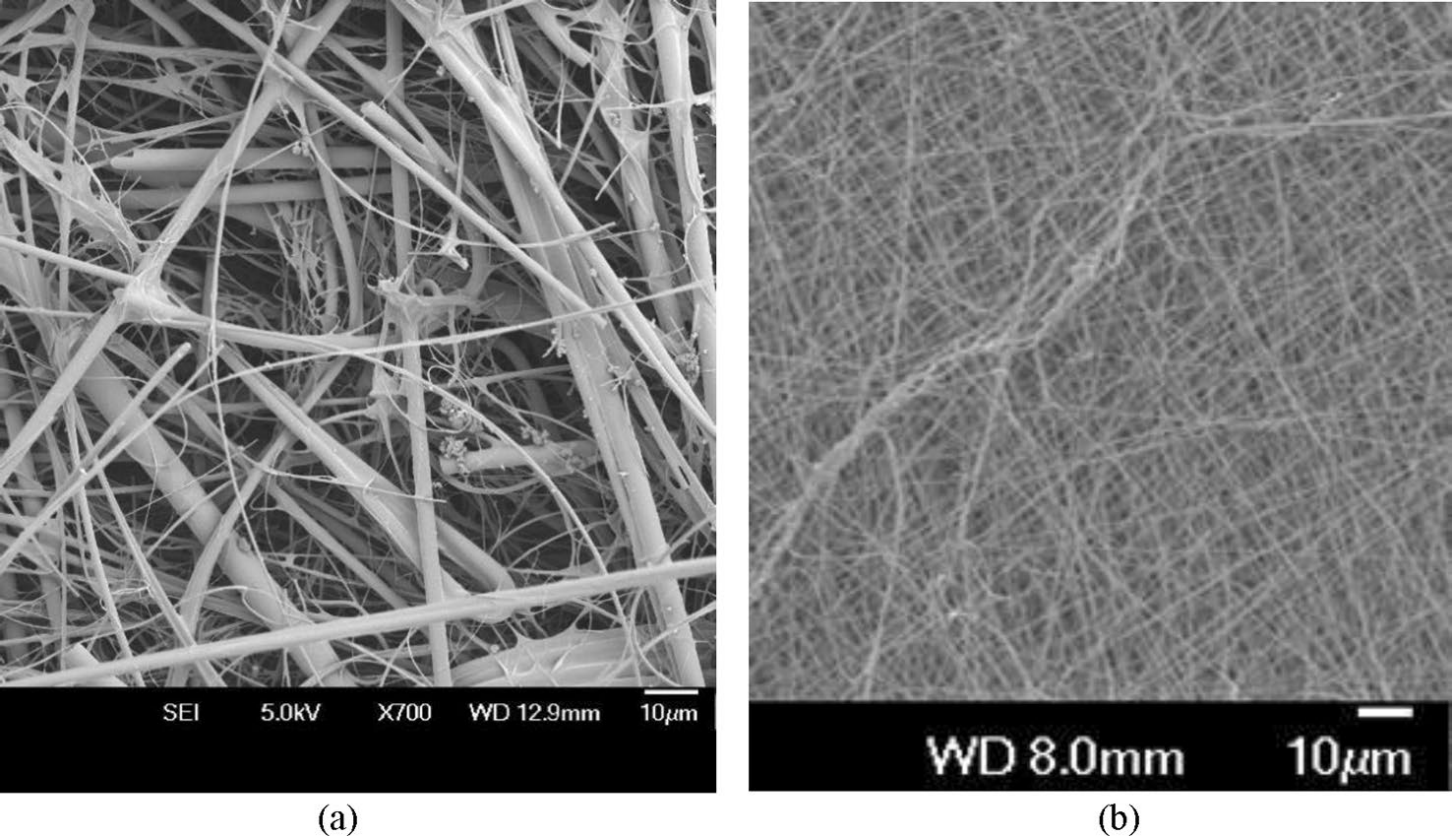
SEM images of filter media. **a** Melt-blown borosilicate glass HEPA filter media. **b** Electrospun and stabilized polyacrylonitrile fiber media at the same level of resolution

Figure 9 shows a comparison of melt-blown glass fiber HEPA filter media with electrospun and stabilized polyacrylonitrile filter media. The glass filter media in Fig. 9a qualifies for nuclear grade HEPA filter media under ASME AG-1 specifications. At the same magnification, the electrospun and stabilized polyacrylonitrile filter media has smaller diameter nanofibers packed more densely. As discussed in Sect. 3.3 and illustrated in Fig. 2 above, the fiber diameter size has a significant impact on the air filtration efficiency and air flow resistance. Media with smaller fiber diameter sizes, such as the polyacrylonitrile fiber media in Fig. 9b, has a higher total filter efficiency than comparable media with larger diameter sizes such as the glass media shown in Fig. 9a. However the polyacrylonitrile fiber media in Fig. 9b would also exhibit a higher resistance to air flow than the glass media of Fig. 9a, which also must be considered. Additional considerations besides the air filtration efficiency and air flow resistance of comparable filter media shown in Fig. 9 include the media overall strength, resistance to tearing, and flexibility.

Continual advancements in glass technologies have created many potential alternatives for HEPA filtration media. Dry-laying and wet-laying glass staple fibers, chopped strands, and yarns are alternative methods to produce filter media. The limitation for advancement of glass media for HEPA filtration is the range of possible diameter sizes.

### Basalt Fiber Nonwoven Media

Basalt fibers can be produced through the same methods as glass fiber production, however, without any additives to the basalt. The abundance of basalt, ease of production, and physical characteristics of basalt fibers make them well suited for high temperature HEPA filtration. Long-term burst testing of filter bags at the Nebraska Power Sheldon Station showed a distinct advantage of the basalt media over other media types (Medvedyev and Tsybulya 2005). The basalt composite filter bags maintained the same strength over several years of exposure to high temperature exhaust air flow above 200 °C while the other media types lost 20% or greater strength capacity in the same conditions (Medvedyev and Tsybulya 2005). Basalt composite filter media is capable of capturing exceedingly hot particles, as in the production of nickel film or molybdenum powder production, where the captured particles often exceed 800 °C (Medvedyev and Tsybulya 2005). Long-term permeability testing of filter bags at the St. Lawrence cement plant in Ontario Canada showed basalt-P84 composite filter bags superior to glass-PTFE composite media for pulse cleaning and re-use (Medvedyev and Tsybulya 2005). These examples illustrate significant viability of basalt filter media for high temperature HEPA filtration.

### Carbon Fiber Nonwoven Media

Many companies commercially produce carbon fiber nonwoven media primarily intended for structural composite reinforcement. Activated carbon media is also available for home and industrial air filtration, however, most activated carbon filters use polymer fibers for filtration and activated carbon only for gas adsorption. Electrospun, stabilized, and carbonized polyacrylonitrile filter media has significant potential for HEPA filtration. Electrospinning has an advantage over melt-blowing in the production of smaller fiber diameter sizes. Electrospun nanometer fibers are well suited for high filtration efficiency and low flow resistance.

### Ceramic Fiber Nonwoven Media

Continual advancement of polymer science and ceramics is widening the potential application of nonwoven ceramics in air filtration. Oxide and non-oxide ceramics can be dissolved into solution and electrospun into nanometer size diameter fibers through the sol–gel process, resulting in nonwoven filter media. Ceramic fibers also do not absorb moisture and easily withstand high temperatures.

Over the past several years Lawrence Livermore National Laboratory developed and tested high strength oxide ceramic air filters intended for nuclear air ventilation (Haslam 2018; Haslam and Mitchell 2013; Kelly et al. Oct. 2018). Oxide ceramic nanofibers, created with electrospinning and thermal heat treatment, can be assembled into nonwoven filter media with mean fiber diameters between 50 and 150 nm (Haslam 2018). Ceramic filter media elements survived exposure to high temperature air flows of 500 °C (Haslam 2018).

Laboratory experiments of filtration efficiency using aluminum oxide-stabilized zirconium dioxide (ASZ) ceramic filter media illustrate the significant potential of ceramic HEPA media (Jia et al. 2020). The ASZ filter paper media, created with a simple solution blow spinning and calcination method, demonstrated flexibility, foldability, and temperature usage capacity up to 1100 °C (Jia et al. 2020). Foldable ceramic fiber nonwoven media such as ASZ might easily form pleated filter paper for use in HEPA units. Another novel design for using ceramic fiber nonwoven media in HEPA applications developed at Lawrence Livermore is the “mini-tubular” construction method (Kelly et al. 2018). The mini-tubular design reduces the problems associated with ceramic filter element shrinkage during production. The mini-tubular ceramic filter concept shows potential for HEPA air filtration.

### Metal Fiber Nonwoven Media

Bekaert designs and produces sintered metallic nonwoven fibrous media intended for use as HEPA filtration (Bekaert 2020). Metal fibers and metallic filtration media easily withstand the strength, durability, flexibility, and high temperature requirements of the ASME AG-1 specifications listed above. The ductility of metallic nonwoven media is noted as a distinct advantage over brittle media, as the metallic media can be shaped and compressed to modify the solidity with less danger of fibers breaking (Manzo et al. 2016). For this reason metallic media is appropriate for intake and exhaust filtration of combustion engines and other machinery that experiences vibration (Manzo et al. Apr. 2016).

Metal fibers do not absorb moisture and resist bacteria growth which is a distinct advantage over moisture-absorbing fiber types. While not absorbing moisture, metal fiber media has characteristically high wettability, making it advantageous for coalescence air filtration (Manzo et al. Apr. 2016; Liew and Conder 1985; Conder and Liew 1989). A direct comparison of Sullube 32 oil droplet aerosol filtration efficiency and air flow resistance between 6.0 μm glass fiber media and 6.5 μm stainless steel fiber media revealed significant performance advantage of the stainless steel media (Manzo et al. Apr. 2016).

The noted challenge in advancing metal fibers for HEPA filtration is the limitation on available metal fiber diameter sizes. Metallic filter media has good potential as a component of composite HEPA filter media, by combining the mechanical advantages of metal media with the smaller diameter size advantage of other media types.

### Polymer Fiber Nonwoven Media

Air filters for personal protective gear and home filter units are made with nonwoven polymers. Polypropylene does not absorb moisture like many other synthetic and natural fibers, and has become the dominant fiber for personal face masks and pleated filter cartridges. Studies indicate melt-blown polypropylene fibers may have a higher quality factor than glass H13 filter paper through dust loading (Hwang et al. 2018). Shanghai Mingguan Purification Materials produces melt-blown polypropylene filter paper for personal protective masks and filter units that achieve H13 and H14 standards (Shanghai Mingguang 2022). However polypropylene fibers cannot withstand the high temperature requirements specified by ASME AG-1 to qualify as a nuclear grade HEPA filter media. Spectra and Dyneema are UHMWPE polymer fibers which don’t absorb moisture, and although not capable of meeting ASME AG-1 specifications because of temperature limitations, may potentially serve as suitable alternatives to polypropylene fibers for use in moderate temperature environments. Polyimide P84 fibers and Nomex aramid fibers are used extensively in industrial air and gas separation bag filter units for cement factories and power plants. These polymer filter materials are also limited to temperatures below the AG-1 specification.

Besides melt blowing, many polymers are well suited for electrospinning into filter media with nanometer scale fiber diameters and uniform fiber shape, resulting in high filtration efficiency (Bonfim et al. 2021; Karabulut et al. 2021; Zhu et al. 2017). Figure 9b shows the smaller fiber diameter sizes and uniformity of fiber shapes compared to the melt-blown borosilicate glass media shown in Fig. 9a. Electrospinning polyacrylonitrile nanofibers into air filter media results in high-filtration efficiency with low flow resistance (Yun et al. 2007). The advantage of electrospun polyacrylonitrile fibers is they can be stabilized carbonized into HEPA filter media that achieves ASME AG-1 standards for high temperature.

## Conclusion

Air filtration began in earnest on the battlefields of the Second World War eighty years ago, relying entirely on natural materials to include cotton, esparto, wool, and asbestos. Significant advancement in air filtration technology over the decades included the replacement of natural materials for safer and more efficient synthetic materials. Melt-blown borosilicate glass has become the fiber of choice for nonwoven HEPA filter units today, while melt-blown polypropylene fibers are the choice for personal protective equipment. However more materials research may advance air filtration technology to increase the efficiency and reduce the flow resistance of HEPA filter units. The research topics shown in Table 13 are recommended for further HEPA air filtration development of alternative filter media.

**Table 13:** Recommended materials research in filter materials

Fiber type	Further research recommendation
Glass	Testing additional glass fiber types (A, C, E, ECR, S2, R, etc.) Production methods to reduce fiber diameter size
Basalt	Production of nonwoven continuous basalt fibers in filter media
Carbon	Electrospinning carbon precursors for air filtration media Carbon nanotube filtration in free molecular flow regime
Ceramics	Electrospinning of sol–gel nonwoven ceramic filter media
Metals	Advancing sintered metal filter media

Any newly developed HEPA filter media must be tested and classified according to, ISO 29463, EN 1822, or ASME AG-1 depending on the particular intended application. In order to qualify a newly developed filter media for use in nuclear grade HEPA filter units, additional testing is required beyond filtration efficiency and flow resistance to ensure the media integrates into the filtration infrastructure and system. For example, the support infrastructure must withstand the weight and vibration requirements of new filter media as it is tested as part of the overarching air filtration system.

## Data Availability

The data presented in this study are available in referenced materials.

## Appendix

See Appendix Tables

**Table 14:** Listing of variables

Term	Description	Unit of measurement
$\mathrm{c}$	Linear density	kg/m
${d}_{\mathrm{f}}$	Fiber diameter	m
*k*	Inverse of radius of curvature	m^−1^
$s$	Fiber shape factor	Dimensionless
$E$	Tensile modulus	Pa
*M*	Moment of inertia	kg·m^2^
${E}_{\mathrm{S}}$	Specific modulus ($E/\uprho$)	Dimensionless
$\mathrm{EI}$	Flexural rigidity of a beam	N·m^2^
${E}_{\mathrm{F}}$	Total filter efficiency	Dimensionless
${E}_{\Sigma }$	Combined single fiber efficiency of components	Dimensionless
${E}_{\mathrm{D}}$	SFE diffusion component	Dimensionless
${E}_{\mathrm{DR}}$	SFE for interception of diffusing particles	Dimensionless
${E}_{\mathrm{E}}$	SFE electrostatic component	Dimensionless
${E}_{\mathrm{G}}$	SFE gravity component	Dimensionless
${E}_{\mathrm{I}}$	SFE inertial impaction component	Dimensionless
${E}_{\mathrm{R}}$	SFE interception component	Dimensionless
$\mathrm{QF}$	Quality factor, figure of merit	Pa^−1^
$t$	Thickness of the air filter media	m
${U}_{0}$	Air velocity	m/s
${\alpha }_{\mathrm{f}}$	Solidity, solid volume fraction, fiber packing density	Dimensionless
$\Delta P$	Pressure differential, flow resistance	Pa
$\eta$	Air viscosity	Pa·s
$\rho$	Volumetric density of the fiber material	kg/m^3^

^14^,

**Table 15:** Listing of derived units of measurement

Unit	Description	SI base units
$\mathrm{tex}$	Tex, measure of linear density	1.0E-6·kg/m
$\mathrm{dtex}$	Decitex, measure of linear density	1.0E-7·kg/m
$\mathrm{de}$	Denier, measure of linear density	9.0E-6·kg/m
$\mathrm{gpd}$	Grams per denier, measure of specific strength	8.826E4·m^2^·s^−2^
$\mathrm{yuri}$	Measure of specific strength	m^2^·s^−2^
$\mathrm{MY}$	Mega Yuri, measure of specific strength	1.0E6·m^2^·s^−2^

^15^

Rapid advancement of the fiber industry creates a challenge to maintain currency with brand names and mechanical characteristics. The data used in this review was compiled from various online sources including commercial brochures and data tables from other authors as shown in Table

**Table 16:** Data sources

Source	Source	Year	Type
Online	Matweb, Wikipedia	2022	All fiber types
Commercial Technical Guides	Dupont Kevlar Aramid Technical Guide	2022	Aramid
	3 M Nextel Ceramic Fibers Technical Reference Guide	2021	Nextel
	Dupont Nomex Technical Guide	2021	Nomex
	J.P. Stevens Handbook	2017	Quartz and Glass
	Saint Gobain Quartzel Datasheet	2021	Quartzel
Commercial Datasheets	AGY, Binani, DSM, Dupont, Hexcel, Hiltex, Honeywell, Mitsubishi,	2021	All fiber types
	Nycon, Solvay, Specialty Materials, Teijin, Texonic, Torray, Toyobo		
Handbooks and Data Books	Handbook of Properties of Textile and Technical Fibers	2018	All fiber types
	Handbook of Nonwoven Filter Media	2016	All fiber types
	Advances in Technical Nonwovens	2016	Cellulose
	Ullmann's Encyclopedia of Industrial Chemistry	2012	Ceramic, Glass
	Fibrous Composite Materials for Civil Engineering Applications	2011	All fiber types
	Natureworks Ingeo Technical Bulletin TB180904	2005	Natural
	Handbook of Materials for Product Design	2001	Glass
	Textile Fibers: A Comparative Overview	2001	Natural and Polymer
	Carbon and High Performance Fibres Directory and Databook	1995	All fiber types
	Handbook of Materials for Product Design	2001	Carbon, Ceramic, Glass
	Military Standard 1944, Polymer Matrix Composites	1985	Carbon, Ceramic, Mineral

16. Compiled data for particular fiber types is shown in Table

**Table 17:** Compiled data for particular fibers

Family	Fiber	Density (g/cm^3^)	Tensile strength (GPa)	Tensile modulus (GPa)	Melting/Decomposing Temp (˚C)	Moisture regain (%)	CTE (10^–6^ K^−1^)	Elongation at break (%)	Diameter (µm)
Cellulose	Oak wood fiber (MatWeb 2021)	0.74	0.090	2.66	250	15.0	4.9	2.0	13
Cellulose	Pine wood fiber (MatWeb 2021)	0.35	0.078	468.48	–	–	–	8.2	–
Cellulose	American Uppers Cotton (Yan 2016)	1.52	0.491	7.60	246	8.5	–	7.0	11
Cellulose	Flax (Araújo 2011)	1.54	0.831	27.72	–	12.4	–	3.0	5
Cellulose	Hemp (Araújo 2011)	1.50	0.705	32.55	–	12.4	–	2.2	–
Cellulose	Jute (Araújo 2011)	1.50	0.465	25.80	–	13.8	–	1.8	8
Cellulose	Ramie (Araújo 2011)	1.50	0.885	21.90	–	–	–	3.7	–
Cellulose	Fortisan, Viscose Rayon (Araújo 2011)	1.50	0.885	24.15	175	16.0	–	6.4	9
Cellulose	TriAcetate fiber (Hearle 2001)	1.32	0.172	4.75	230	4.0	–	30.0	15
Cellulose	Tenasco Rayon (Araújo 2011)	1.50	0.405	9.00	175	13.0	–	20.0	12
Keratin	Wool (Huson 2018)	1.31	0.185	5.11	222	16.0	9.2	35.0	18
Keratin	Silkworm silk (Hearle 2001)	1.34	0.509	9.78	175	10.0	–	23.0	10
Keratin	Spider silk (Ko and Wan 2018)	1.31	1.400	–	–	10.0	–	23.0	2
Polymer	Toyobo Zylon HM PBO (Toyobo 2021)	1.56	5.800	270.00	650	0.6	– 6	2.5	1.18
Polymer	Nylon 6 (Teijin 2022)	1.13	0.900	3.00	223	4.5	6.5	25.0	14
Polymer	Nylon 6.6 (Teijin 2022)	1.13	0.900	5.00	260	6.0	6.5	25.0	14
Polymer	Polypropylene (PP) (Araújo 2011)	0.91	0.591	6.46	100	1.0	–	20.0	38
Polymer	Polyacrylonitrile (PAN) (Teijin 2022)	1.40	0.300	11.00	327	10.0	–	23.0	–
Polymer	Polytetrafluoroethylene (PTFE) (Hutten 2015)	2.22	0.392	199.00	–	–	–	25.0	–
Polymer	Polybenzimidazole (PBI) (Teijin 2022)	1.43	0.320	5.10	450	15.0	–	27.0	–
Polymer	Vectran HT Polyester (MatWeb 2021)	1.41	3.200	75.00	–	0.1	–	3.8	–
Polymer	P84 Polyimide (Starr 1995)	1.41	0.540	5.00	–	-	–	30.0	–
Polymer	Polyvinyl alcohol (PVA) (Araújo 2011)	1.30	1.600	22.00	–	–	–	–	–
Polymer	Acrylic (Hearle 2001)	1.19	0.321	7.38	235	1.5	–	28.0	12
Polymer	Toyobo Tsunooga (Toyobo 2021)	0.97	1.400	43.00	145	–	–	6.0	–
Polymer	Akzo Nobel M5 PIPD(Cunniff et al. 2002)	1.70	3.960	271.00	–	–	–	1.4	–
Polymer	Polyester HT (Araújo 2011)	1.39	0.778	18.35	–	–	–	–	–
Aramid	Dupont Kevlar 49 (Dupont 2017)	1.44	3.000	112.40	427	3.5	– 4.86	2.4	12
Aramid	Dupont Kevlar 29 (Dupont 2017)	1.44	3.617	62.00	427	3.5	–	4.0	–
Aramid	Dupont Nomex 430 Meta-aramid (Dupont 2017)	1.38	0.609	8.30	350	5.1	–	30.5	–
Aramid	Teijin Technora (Teijin 2022)	1.39	3.400	74.00	500	1.9	–	4.5	–
Aramid	Teijin Twaron (Teijin 2022)	1.45	3.600	120.00	500	5.0	–	4.4	–
Aramid	Dupont Kevlar 149 (Dupont 2017)	1.47	3.450	179.00	427	3.5	–	–	–
UHMWPE	DSM Dyneema SK99 (DSM 2021)	0.97	4.100	155.00	144	0.0	− 12	4.0	12
UHMWPE	Honeywell Spectra 1000–75 (Honeywell 2021)	0.97	3.680	133.00	147	–	− 12	2.9	1.665
Carbon	Single Wall Carbon Nanotube (Araújo 2011)	1.40	53.000	5000.00	–	–	–	16.0	–
Carbon	Toray T1100G (IM, PAN) (Toray Composite Materials America 2022)	1.79	7.012	324.00	3500	–	– 0.5	2.0	5
Carbon	Toray M-46 J (HM, PAN) (Toray Composite Materials America 2022)	1.84	4.200	436.00	3500	–	− 0.9	1.0	5
Carbon	Solvay P-25 (pitch) (Solvay 2021)	1.95	1.380	159.00	254	–	-0.6	0.5	11
Glass	AR1 glass fiber (Jones and Huff 2018)	2.74	3.240	73.00	1290	–	6.5	–	–
Glass	A glass High Alkali (Jones and Huff 2018)	2.46	3.100	72.00	1600	–	9	4.8	–
Glass	C glass Sodium Borosilicate (Jones and Huff 2018)	2.49	3.400	69.00	800	–	7.1	4.4	3.8
Glass	D glass (Jones and Huff 2018)	2.10	2.400	52.00	–	–	2.5	–	–
Glass	E glass Alumino Borosilicate (Jones and Huff 2018)	2.53	3.700	76.00	1140	-	5.4	4.8	3
Glass	Binani SE1500 ECR (Industries 2022)	2.62	2.500	81.00	–	–	6.1	–	17
Glass	R glass (Jones and Huff 2018)	2.55	4.500	85.00	–	–	4.1	–	–
Glass	S glass Owens Corning (Starr 1995)	2.55	3.450	86.00	–	–	–	–	–
Glass	AGY S1 glass (AGY 2021)	2.52	3.135	90.00	–	–	–	–	–
Glass	AGY ZenTron S2 Magn Borosil (MatWeb 2021)	2.46	4.890	86.90	816	–	1.6	5.7	7
Glass	AGY S3 glass (AGY 2021)	2.83	3.338	99.00	–	–	–	–	–
Quartz	Saint-Gobain Quartzel (SiO2) (Saint-Gobain 2022)	2.20	6.000	78.00	1220	–	0.54	7.7	9
Quartz	J.P. Stevens Astroquartz II (SiO2) (JPS 2022)	2.20	6.000	72.00	1120	–	–	–	–
Ceramic	Specialty Materials SCS Ultra SiC (Specialty Materials 2021)	3.08	5.900	415.00	1550	–	4.1	0.9	75
Ceramic	3 M Nextel 610 alumina silica (3M 2022)	3.90	2.800	370.00	2000	–	8	–	10
Ceramic	Alumina Saffil (MatWeb 2021)	3.30	2.000	300.00	2000	–	5.2	–	3
Ceramic	Specialty Materials SCS6 SiC (Specialty Materials 2021)	3.08	3.900	380.00	–	–	–	–	–
Ceramic	3 M Nextel 440 (3M 2022)	3.00	1.840	190.00	1800	–	5.3	–	–
Ceramic	3 M Nextel 720 (3M 2022)	3.40	1.940	250.00	–	–	–	–	–
Ceramic	3 M Nextel 312 Alumina (3M 2022)	2.80	1.630	150.00	–	–	–	–	–
Mineral	Basalt fiber (Texonic 2021)	2.64	4.137	84.80	1450	3.0	8	3.0	10
Mineral	Specialty Materials Boron 4mil (Specialty Materials 2022)	2.61	4.000	428.00	1350	–	4.5	–	102
Metal	Inconel 601GC Nickel Superalloy (MatWeb 2021)	8.11	0.790	206.50	1360	–	13.75	55.0	–
Metal	Stainless steel (Wikipedia 2021)	8.00	0.505	70.40	1370	–	–	–	–
Metal	Brass fiber (Wikipedia 2021)	8.55	0.580	76.95	1260	–	–	–	–
Metal	Copper (Wikipedia 2021)	8.92	0.220	1516.40	1293	–	–	–	–
Metal	Aluminum (Araújo 2011)	2.70	0.620	70.00	–	–	–	–	–

17.
